# Spontaneous porto‐femoral shunting in long‐standing portal hypertension

**DOI:** 10.1002/ccr3.2947

**Published:** 2020-05-25

**Authors:** Mauro Giuffrè, Paola Martingano, Lory Saveria Crocè

**Affiliations:** ^1^ Department of Medical Surgical and Health Sciences University of Trieste Trieste Italy; ^2^ Italian Liver Foundation Trieste Italy; ^3^ Department of Radiology Azienda Sanitaria Universitaria Giuliano‐Isontina Trieste Italy; ^4^ Liver Clinic Azienda Sanitaria Universitaria Giuliano‐Isontina Trieste Italy

**Keywords:** liver cirrhosis, paraumbilical vein, portal hypertension, spontaneous portosystemic shunt

## Abstract

Spontaneous portosystemic shunting is a compensation mechanism that is supposed to relieve the portal circulation from high pressures. Here we report an unusual shunt that originates from a patent paraumbilical vein and reaches the femoral vein via the inferior epigastric vein. Despite being merely anecdotal, this finding is fascinating from an anatomical point of view.

## Graphical abstract

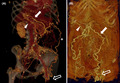

A 78‐year‐old woman with a known history of decompensated alcohol‐related cirrhosis and portal hypertension underwent a contrast‐enhanced CT scan after a focal liver lesion was found on screening ultrasonography. The CT scan identified the lesion as a benign vascular anomaly and revealed an unusual portosystemic shunt (Figure 1): a patent paraumbilical vein, which drained into the systemic circulation via the femoral vein. Generally, paraumbilical veins originate from the left portal vein, run through the falciform ligament, extend toward the umbilicus, and drain into the systemic circulation via the inferior epigastric vein, which was known to reach as far as into the external iliac vein.

**Figure 1:**
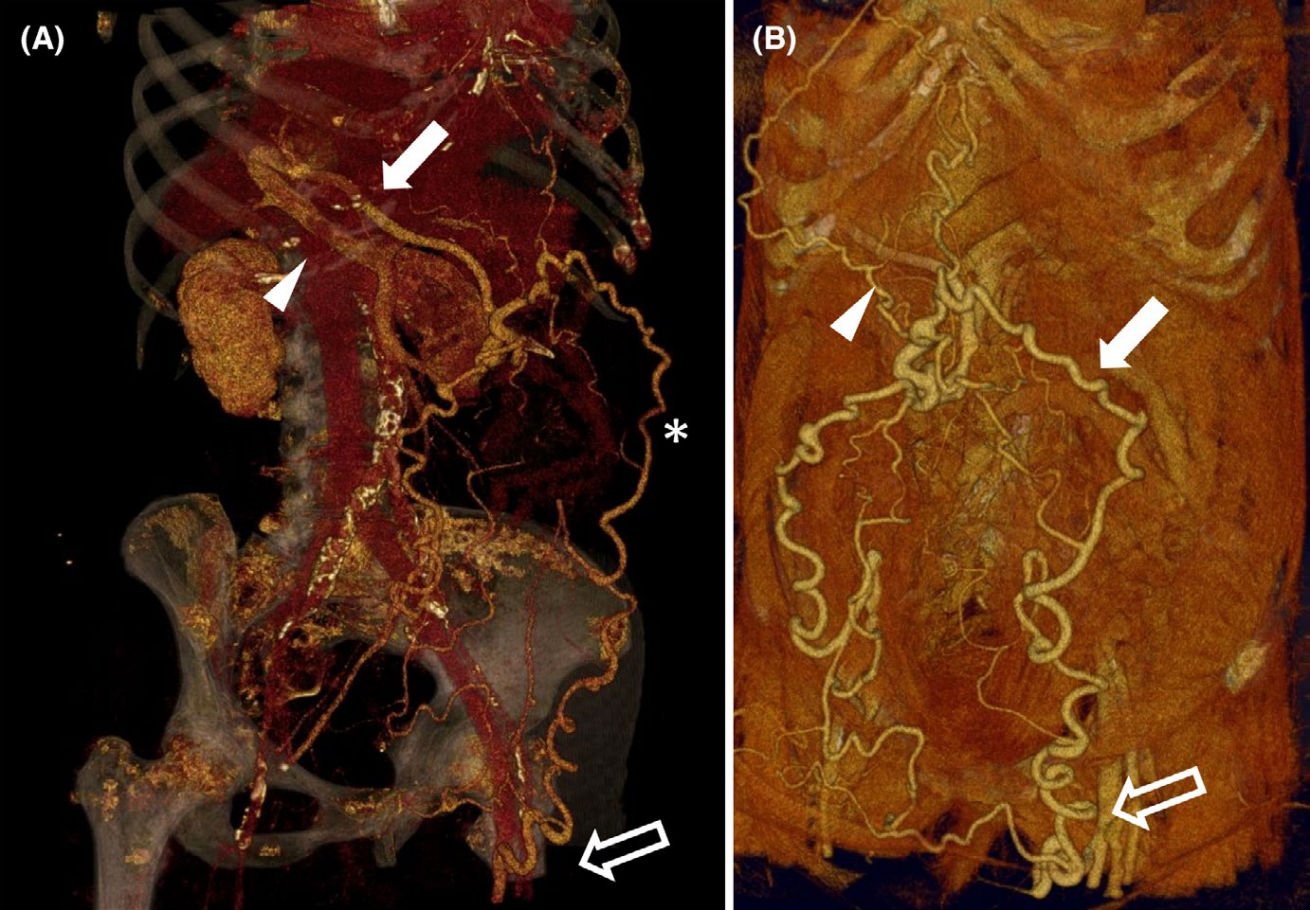
The venous shunting pathway of the patient is shown in the figure panels obtained through volume rendering 3D reconstruction: (A) A dilated portal vein (arrowhead) that communicate throughout patent paraumbilical vein (arrow) with subcutaneous periumbilical veins and inferior epigastric vein (*), with eventual spontaneous femoral vein shunt (empty arrow). B, A patent paraumbilical vein (arrowhead) with subcutaneous periumbilical veins and inferior epigastric vein (arrow), with eventual spontaneous femoral vein shunt (empty arrow)

In portal hypertension, the recanalization of dormant collaterals is a compensation mechanism that allows the decompression of portal circulation, via the diversion of blood flow to the systemic circulation.^1^ In particular, shunting through paraumbilical veins can be associated with the clinical manifestation of *caput medusae* in the epigastric region or herniation of umbilical varices,^2^ which were not present on physical examination. Besides, Doppler examination of the lower limb did not reveal any flow anomaly or signs of superficial/deep vein thrombosis.

In conclusion, this uncommon finding is merely anecdotal but fascinating from an anatomical point of view.

## CONFLICT OF INTEREST

The Authors declare no conflict of interest.

## AUTHOR CONTRIBUTIONS

MG and PM: discovered the radiological finding; PM: performed CT scan volume rendering 3D reconstruction; MG, PM, and LSC: drafted the manuscript; MG, PM, and LSC: approved the final version of the manuscript.

## References

[1] Simón‐Talero M , Roccarina D , Martínez J , et al. Association between portosystemic shunts and increased complications and mortality in patients with cirrhosis. Gastroenterology. 2018;154(6):1694‐1705.e4. 29360462 10.1053/j.gastro.2018.01.028

[2] Morin C , Lafortune M , Pomier G , et al. Patent paraumbilical vein: anatomic and hemodynamic variants and their clinical importance. Radiology. 1992;185:253‐256. 1523319 10.1148/radiology.185.1.1523319

